# DELAYED RETIREMENT IN ISRAEL: FACTORS THAT CONTRIBUTE TO THE NEW POLICY SUPPORT

**DOI:** 10.1093/geroni/igad104.3003

**Published:** 2023-12-21

**Authors:** Polina Ermoshkina

**Affiliations:** Case Western Reserve University, Cleveland, Ohio, United States

## Abstract

Policies that defer retirement age are fiscally justifiable, but not unanimously supported by the citizens. Studies in other countries (United Kingdom, Sweden, and Norway) show that occupational environment (feeling unappreciated at work, having a physically demanding job) affects workers’ support for pension reform. Israel is a unique national context for this question because of the high proportion of immigrants who had not spent their working lives in Israel. This study used The Survey of Health, Ageing and Retirement in Europe (SHARE) Israel (2005-2006) to examine how the current job conditions (having a physically demanding job, having a job that allows workers to develop new skills, receiving support from the supervisors, job satisfaction) affect Israeli workers’ attitudes towards delayed retirement age. The analysis was limited to 482 respondents. The average age of the participants was 64.1 (SD=10.1) years. The participants included veteran Jewish-Israelis, Arab-Israelis, and new immigrants from the former Soviet Union. Nearly 40 percent of the respondents were very interested in working until the new retirement age yet 24.73% were not interested at all. Multiple correlation showed that low job satisfaction, having a physically demanding job, and having a job that does not allow one to develop new skills were associated with low interest in prolonged retirement age. Gender, birthplace, and having adequate support at work were not associated with workers’ support for delayed retirement. These findings indicate that older adults in demanding, low-reward work most strongly oppose extended working life.

